# Clinical outcomes of quad-loop intraocular lenses: trifocal, bifocal and toric types

**DOI:** 10.5935/0004-2749.2025-0085

**Published:** 2025-09-10

**Authors:** Camila Mendes Costa Campelo, Fabricio Afonso Borges Silva, Paula Marques Marinho, Diego Casagrande, Allexya Affonso Antunes Marcos, Lincoln Lemes Freitas, Milton Seiyu Yogi, Rubens Belfort Junior

**Affiliations:** 1 Department of Ophthalmology, Instituto Paulista de Ensino e Pesquisas em Oftalmologia, São Paulo, Brazil; 2 Department of Ophthalmology, Universidade Federal de São Paulo, São Paulo, Brazil

**Keywords:** Cataract extraction, Aberrometry/methods, Lenses, intraocular, Lens implantation, intraocular, Prosthesis design

## Abstract

**Purpose:**

The purpose of this study was to assess visual outcomes and patient satisfaction following cataract surgery involving the implantation of quad-loop intraocular lenses, including trifocal, bifocal, and toric variants.

**Methods:**

Information was obtained from both physical and electronic medical records of patients who underwent phacoemulsification cataract surgery with implantation of different intraocular lenses between January 1, 2022, and December 31, 2023. The study included individuals aged over 18 who received bilateral implantation of bifocal, trifocal, or monofocal toric intraocular lenses. Visual acuity was assessed at various postoperative time points using the logMAR scale. Quantitative variables were analyzed using mean and standard deviation.

**Results:**

A total of 92 eyes received premium intraocular lenses: 4 bifocal, 32 trifocal, 52 toric monofocal, and 4 trifocal toric lenses. The average preoperative corrected visual acuity was logMAR 0.478 ± 0.259. On the first postoperative day, the average uncorrected visual acuity was logMAR 0.301 ± 0.207. By day 30, 67.4% of eyes achieved uncorrected distance visual acuity of logMAR 0.2 or better. Patient satisfaction was high, with few reports of glare or halos.

**Conclusion:**

Quad-loop intraocular lenses-including trifocal, bifocal, and toric models-demonstrated effective improvement in visual acuity and high levels of patient satisfaction. These lenses represent a suitable option for enhancing visual outcomes after cataract surgery. Additional studies with larger cohorts are recommended to confirm these results.

## INTRODUCTION

In recent years, cataract surgery has seen notable progress, particularly with the development of various intraocular lens (IOL) options aimed at enhancing visual outcomes for patients. Among these, toric, bifocal, and trifocal IOLs have gained popularity, each offering specific advantages and associated considerations^(1)^.

Toric IOLs are specifically engineered to correct astigmatism, a prevalent refractive error. Research has shown that these lenses can deliver excellent distance visual acuity and lessen the reliance on corrective eyewear following cataract surgery^(2)^. Achieving the best results, however, requires meticulous preoperative planning and precise intraoperative alignment of the lens.

In contrast, bifocal and trifocal IOLs provide the added advantage of enhancing near and intermediate vision, thereby decreasing the need for reading glasses. While these multifocal lenses have been associated with improvements in patients’ perceived quality of vision, they may also lead to certain visual effects, including reduced contrast sensitivity, glare, and halos. Despite these potential drawbacks, most patients fitted with multifocal lenses report high satisfaction with their visual outcomes^(2^,^3)^.

Careful patient selection and thorough preoperative counseling are essential to ensure that individual visual needs and expectations are consistent with the capabilities of the selected IOLs. Additionally, comprehensive preoperative evaluation of the ocular surface and macula, along with precise surgical execution, are key components for achieving favorable outcomes with advanced IOLs^(2)^. Current evidence indicates that toric, bifocal, and trifocal IOLs are generally safe and effective options for cataract surgery, as long as patients are appropriately evaluated and the selected lens is correctly implanted^(2^,^4^,^5)^.

Achieving accurate lens positioning is critical for maximizing visual acuity and minimizing postoperative complications. Misalignment of the lens can result in various issues, such as residual refractive errors, visual aberrations, and reduced visual performance^(6)^. The quad-loop IOL represents a variation of the conventional single-piece IOL design, incorporating four peripheral loops rather than the typical three. This distinct structural design is thought to provide several advantages, including enhanced stability within the capsular bag, reduced tilt and decentration, and potentially improved optical outcomes^(7)^.

Given ongoing advancements in IOL technology and the development of new designs, further studies are necessary to evaluate their actual benefits in terms of patient satisfaction, visual acuity, refractive predictability, visual quality, and the perception of optical phenomena. The objective of this study was to evaluate the safety and initial outcomes associated with this type of IOL in cataract surgery.

## METHODS

This observational, retrospective study is based on data collected from both physical and electronic medical records via the VECTOR system. It includes patients who underwent cataract surgery through phacoemulsification with implantation of bifocal non-toric, trifocal toric, trifocal non-toric, and monofocal toric IOLs at the *Instituto Paulista de Ensino e Pesquisas em Oftalmologia* (IPEPO)–*Instituto da Visão*, between January 1, 2022, and December 31, 2023. The study is registered under ethics committee approval number 61289622.0.0000.0082. All patients undergoing phacoe mulsification with high-technology IOL implantation at the institution will be invited to participate and will sign the Free and Informed Consent Form, authorizing the use of their data for research purposes.

### Patients

Inclusion criteria consisted of individuals over the age of 18 who underwent phacoemulsification surgery with bilateral implantation of bifocal, trifocal, or monofocal toric IOLs during the specified study period and who were able to provide informed consent and understand the study information.

Exclusion criteria included the presence of significant irregular corneal astigmatism, diagnosis of severe degenerative visual conditions (e.g., macular degeneration or other retinal disorders), history of corneal surgery, amblyopia, clinically relevant corneal endothelial dystrophy (e.g., Fuchs dystrophy), prior corneal diseases (e.g., herpes simplex, herpes zoster, etc.), diabetic retinopathy, history of retinal detachment, glaucoma, and enrollment in any other study involving ocular surgical procedures.

After selecting eligible participants, a comprehensive evaluation was conducted to collect the data required to determine the most suitable IOL for each patient. Biometric analysis was performed using the Zeiss IOLMaster 500 optical biometry, which measured key ocular parameters including axial length and corneal curvature^(8^,^9)^. The posterior segment of the eye was assessed with the Heidelberg Engineering SPECTRALIS OCT system, which produced detailed retinal images and helped identify any underlying pathologies not detected during fundoscopy^(9^,^10)^. Corneal surface mapping was then completed using the EyeSys Vista Topography Systems to generate a detailed corneal topography profile.

Following these examinations, IOL power was calculated using the Barrett II and Barrett True K formulas to determine the most appropriate lens for each patient^(11)^. The Barrett II formula is particularly effective in incorporating variables such as anterior chamber depth, lens thickness, and corneal power, all of which can significantly influence the final refractive result. Likewise, the Barrett True K formula offers a more precise calculation of true corneal power, which is especially important when selecting toric IOLs for the correction of corneal astigmatism^(11)^. Based on these calculations, along with consideration of each patient’s daily activities, lifestyle, and personal preferences, the most suitable IOL was selected for each case.

### IOLs studied

**Figure 1:**
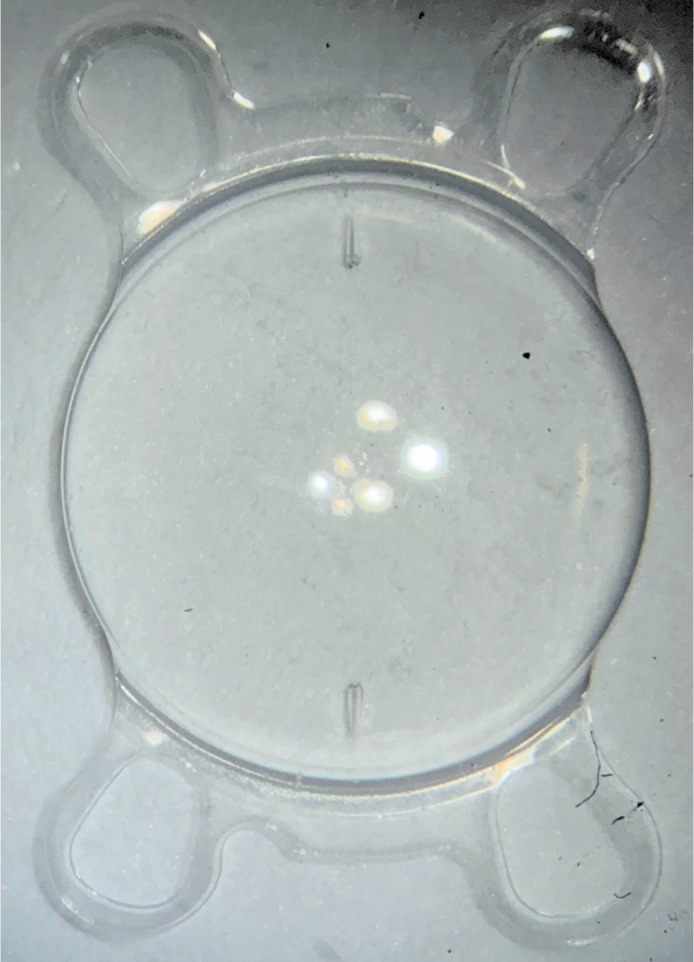
Leedsay AT Toric intraocular lens.

This study evaluated the performance of IOLs featuring a quad-loop haptic design, illustrated in figure 1 by the Leedsay AT Toric monofocal toric lens. Three specific models were included in the analysis. The Leedsay AMF Trifocal is a foldable, hydrophilic acrylic IOL with a diffractive multifocal design and a quad-loop haptic configuration. It incorporates 11 diffractive rings, with an intermediate add power of +1.75 D and a near add power of +3.50 D. The Leedsay AMF Bifocal is likewise a foldable, hydrophilic acrylic diffractive multifocal IOL with a quad-loop haptic structure. This model contains 14 diffractive rings and provides a near add power of +3.00 D. The Leedsay AT Toric is an aspheric monofocal toric IOL composed of hydrophilic acrylic material, also incorporating a quad-loop haptic design.

### Evaluated data and statistical analysis

Data collected from patients’ medical records included binocular visual acuity measured in logMAR; manual refraction using the Early Treatment Diabetic Retinopathy Study chart; near vision assessment with the Jaeger reading chart; anterior segment examination with slit lamp biomicroscopy, including assessment of lens position such as IOL decentration and tilt; intraocular pressure measured by applanation tonometry; corneal topography; and indirect fundoscopy findings, including retinal mapping and macular imaging with optical coherence tomography (OCT-M).

These measurements were recorded at four different visits: preoperative, 1 day postoperative, 1 month postoperative, and 2 months postoperative. Quantitative variables were summarized using mean and standard deviation, while qualitative variables were presented as absolute numbers (n) and relative frequencies (%). The prevalence of satisfactory visual acuity was determined along with its 95% confidence interval (95% CI).

## RESULTS

A total of 92 eyes received premium IOLs (IOL), of which 4 (4.45%) were bifocal lenses, 32 (35.56%) were trifocal IOLs, 52 (56.52%) were monofocal toric IOLs, and 4 (4.45%) were trifocal toric lenses.

The mean preoperative corrected visual acuity was logMAR 0.478 ± 0.259, with values ranging from a minimum of logMAR 1.1 to a maximum of logMAR 0.2.

On postoperative day 1, seven eyes (7.06%) were not tested for visual acuity due to corneal edema. Among the remaining 85 eyes (92.3%), the mean uncorrected visual acuity on the logMAR scale was 0.301 ± 0.207. Table 1 provides a summary of the mean distance visual acuity on the logMAR scale.

**Table 1:** Visual acuity results according to the type of intraocular lens

IOL implanted	CDVA Preop (logMAR)	UDVA PO1 (logMAR)	UDVA PO30 (logMAR)	CDVA PO30 (logMAR)
**Bifocal**	0.45 ± 0.08	0.07 ± 0.04	0.07 ± 0.04	0.05 ± 0.05
**Trifocal**	0.49 ± 0.21	0.30 ± 0.27	0.14 ± 0.18	0.11± 0.14
**Trifocal toric**	0.65 ± 0.39	0.33 ± 0.17	0.23 ± 0.18	0.13 ± 0.12
**Monofocal toric**	0.47 ± 0.29	0.31 ± 0.3	0.2 ± 0.24	0.11 ± 0.13

IOL= intraocular lens; CDVA= corrected distance visual acuity, logMAR= logarithm of the minimum angle of resolution; UDVA= uncorrected distance visual acuity; PO1= postoperative day 1; PO30= postoperative day 30.

At the 30-day postoperative evaluation, three patients were excluded due to missed appointments. Among the remaining 89 eyes, the mean uncorrected distance visual acuity (UDVA) was logMAR 0.18 ± 0.23, with 60 eyes (67.4%) achieving a UDVA of logMAR 0.2 or better. Within this group, 17 eyes (19.1%) had a UDVA of logMAR 0, 27 eyes (30.33%) had logMAR 0.1, and 14 eyes (15.73%) had a UDVA of logMAR 0.2. Corrected visual acuity on postoperative day 30 was logMAR 0.111 ± 0.13, improving further to logMAR 0.03 ± 0.06 by postoperative day 60.

On postoperative day 30, 2 eyes (3.57%) of the 56 implanted with toric IOLs (both toric trifocal and monofocal toric) showed slight temporal displacement; however, this did not affect visual quality or cause reports of glare or halos when patients were questioned. Of the total 92 eyes implanted with bifocal, trifocal, and toric multifocal IOLs, 2 eyes (2.22%) from the same patient reported experiencing glare and halos when inquired.

Among the 40 eyes implanted with multifocal lenses, 31 (77.5%) achieved uncorrected near vision of J2 or better, with 18 (45%) reaching J1 near vision at the 30-day postoperative evaluation. All patients with multifocal lenses who reached the 60-days postoperative follow-up attained corrected near vision of J2 or better.

## DISCUSSION

This study has aimed to assess visual outcomes and patient satisfaction after the implantation of quad-loop IOLs, including trifocal, bifocal, and toric models. The results from the detailed evaluation of multiple parameters offer important insights into the effectiveness and performance of these premium IOLs^(2^,^3^,^7)^.

By including bifocal, trifocal, and toric lenses in the analysis, the study provided a broad understanding of their individual advantages and limitations. The varied patient population, primarily those undergoing cataract surgery with phacoemulsification, also enhances the applicability of the findings.

The assessment of visual acuity at various postoperative intervals showed encouraging outcomes. Most eyes exhibited notable improvement in UDVA 1 day after surgery, with continued progress seen at 30 and 60 days postoperatively. The proportion of eyes achieving satisfactory UDVA, defined as logMAR 0.2 or better, steadily increased during follow-up, reaching 67.4% by the 30-day visit. This gain in uncorrected visual acuity underscores the effectiveness of the IOLs evaluated in providing refractive correction.

Evaluating visual disturbances such as glare and halos is essential to understand the overall quality of vision after surgery^(2^,^7)^. Although only a small number of patients reported these symptoms, their occurrence highlights the need for careful patient counseling about possible visual side effects associated with multifocal lenses. Nevertheless, the overall effect on patient satisfaction and visual performance appears limited, with most patients achieving uncorrected near vision of J2 or better.

The minor temporal displacement observed in a small number of toric IOLs did not have a significant impact on visual outcomes or cause visual disturbances. These results indicate the stability and relaiability of toric IOLs in correcting astigmatism while maintaining optical quality.

In conclusion, the trifocal, bifocal, and toric quad-loop IOLs showed positive results regarding visual acuity, refractive accuracy, and patient satisfaction. The detailed evaluation of various factors, including visual phenomena and refractive outcomes, provides a comprehensive understanding of the performance of these lenses^(2^,^3)^. Although additional studies with larger populations and longer follow-up are needed for further validation, the findings of this study support the use of quad-loop IOLs as an effective option for cataract surgery patients, improving visual results and quality of life.

## Data Availability

The contents underlying the research text are included in the manuscript.
